# Potential Therapeutic Targets for the Treatment of HPV-Associated Malignancies

**DOI:** 10.3390/cancers16203474

**Published:** 2024-10-14

**Authors:** Ziyao Lu, Shahab Haghollahi, Muhammad Afzal

**Affiliations:** Dartmouth Cancer Center, Manchester, NH 03104, USAmuhammad.z.afzal@hitchcock.org (M.A.)

**Keywords:** HPV-associated carcinoma, E6/E7 protein, cellular therapy, immune vaccines, immunotherapy

## Abstract

**Simple Summary:**

HPV-associated cancers, including cervical cancers and head and neck squamous cell cancers, are increasing in incidence worldwide. HPV cancers present distinct and unique targets for treatment due to specific viral proteins driving oncogenesis. In this review article, we discuss developing treatment modalities for HPV-related cancers, including immune therapies and vaccines.

**Abstract:**

This review article aims to summarize broadly recent developments in the treatment of HPV-associated cancers, including cervical cancer and head and neck squamous cell carcinoma. Relatively new treatments targeting the key HPV E6 and E7 oncoproteins, including gene editing with TALENs and CRISPR/Cas9, are discussed. Given the increased immunogenicity of HPV-related diseases, other therapies such as PRR agonists, adoptive cell transfer, and tumor vaccines are reaching the clinical trial phase. Due to the mechanism, immunogenicity, and reversibility of HPV carcinogenesis, HPV-related cancers present unique targets for current and future therapies.

## 1. HPV and Carcinogenesis

### 1.1. HPV Structure and Subtypes

Human papillomaviruses (HPVs) are small, non-enveloped double-stranded DNA viruses. HPV is surrounded by an icosahedral capsid of two structural proteins, major protein L1 and minor protein L2 [1]. The HPV genome, about eight kilobases long, contains eight open reading frames (ORFs) organized into three parts. These include the early (E) region, which produces proteins E1-E7 that help regulate infected cells; the late (L) region, which makes the structural proteins L1 and L2; and the long control region (LCR), which controls viral DNA replication and gene expression [2]. More than 200 HPV genotypes have been identified and are divided into five genera. Certain genotypes (HPV 16, 18, 31, 33, 35, 39, 45, 51, 52, 56, 58, and 59) in the *alpha* genus are associated with higher risks of developing malignancy (group 1 carcinogens) [3]. HPV16 and 18 in particular are frequently associated with higher rates of cervical cancer, and HPV16 is highly implicated in anal and oropharyngeal cancer [4]. Low-risk HPV subtypes are associated with benign epithelial lesions such as warts that rarely progress to cancer [5].

### 1.2. HPV Carcinogenesis

**Figure 1:**
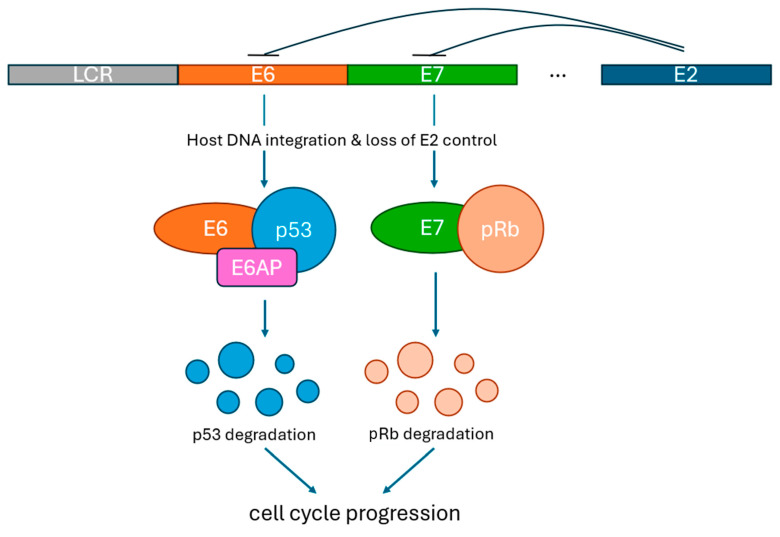
The role of E6 and E7 in loss of cell cycle control. *LCR*—Long control region. *E6AP*—E6 associated protein.

Key viral oncoproteins are responsible for HPV-related carcinogenesis [6]. Continued expression of HPV E6 and E7 proteins has been shown to play a critical role in initiating and maintaining malignancy in HPV-positive cervical cancer cells [7] (Figure 1). E6 and E7 oncoproteins inactivate the p53 and pRb tumor suppressor pathways, respectively. E6 interacts with the ubiquitin ligase E6AP, causing polyubiquitination of p53 and targeting p53 for proteasomal degradation [8]. E7 also binds to p21 and p27, which are involved in cell cycle regulation, removing checkpoint brakes for cellular progression from G1 to S phase [9]. Integration of the initially episomal HPV viral genome into host cell DNA leads to dysregulated expression of E6/E7 through disruption of E2, which normally regulates their expression [10,11]. HPV E5 protein may also be implicated in early carcinogenesis [12]. HPV E6 and E7 proteins present distinct molecular targets given this key role in developing and sustaining carcinogenesis.

## 2. HPV Therapeutic Pathways

HPV-associated cancers include cervical cancer, oropharyngeal cancers, anal squamous cell carcinoma, and vulvar/vaginal cancers. For most HPV-associated cancers, chemotherapy and immunotherapy are standards of care. Beyond conventional chemotherapy and immune checkpoint inhibitors, HPV oncogenes are emerging as targets for new therapies such as therapeutic cancer vaccines, PRR agonists, cellular therapies, and gene editing [13].

## 3. Therapeutic Cancer Vaccines

The goal of therapeutic cancer tumor vaccination is to elicit an adaptive immune response against tumor-associated or tumor-specific antigens (TAA/TSAs) [14]. TAAs such as CEA and HER2 are overexpressed in tumors but can be expressed in normal tissues, whereas TSAs are neoantigens expressed solely by tumors [15]. In HPV-related cancers, E6 and E7 oncoproteins are unique TSAs that represent near-ideal therapeutic targets for vaccination because of their constitutive expression in tumors and key role in oncogenesis. Although no therapeutic HPV vaccinations have been approved, multiple clinical trials have investigated therapeutic vaccinations using different antigen delivery systems [14]. These include E6/E7 DNA, RNA, and peptide-based vaccines, which may be injected intramuscularly, subcutaneously, or intravenously, as well as autologous cell-based vaccines which are harvested from patient immune cells that are exposed to antigens ex vivo and subsequently re-infused into patients [16].

Overall vaccine efficacy appears to be limited based on early Phase I–II clinical trials due to poor immunogenicity and stability in vivo [17]. For instance, three peptide-based vaccine trials showed an overall response rate (ORR) of 0% when used alone. Vaccines are often paired with immune checkpoint inhibitor therapy to prevent cancer immune evasion and potentiate vaccine efficacy. For example, the GX-188E vaccine, which induces E6- and E7-specific T cell response, showed ORR ranging from 33% to 42% when it was combined with pembrolizumab. The ISA101 peptide-based vaccine, which consists of E6 and E7 synthetic long-chain peptides, showed complete response rates (CRR) of 8 to 15% when it was combined with nivolumab. However, the extent to which the efficacy can be attributed to the therapeutic vaccine is unclear, as these outcomes are largely similar to those of trials that studied immune checkpoint inhibitors alone in comparable patient groups.

To improve vaccine efficacy, other delivery modalities have been studied. More recently, mRNA vaccines have gained traction in the context of the SARS-CoV-2 pandemic, largely due to advances in lipid nanoparticle (LNP) technology, which have enabled the development of more stable mRNA-LNP constructs with increased immunogenicity. Previous mRNA vaccines have had limited efficacy due to rapid mRNA degradation during delivery to target cells [18]. New mRNA-LNP vaccines have shown promising results in mouse models [19] against HPV-positive oropharyngeal cancer and cervical cancer [20]. Moreover, immunization of rhesus monkeys with these mRNA-LNP vaccines targeting E6 and E7 antigens have shown promising immunogenicity [19], further providing strong evidence for possible clinical application against HPV-related cancers in humans. Future clinical trials are needed to evaluate the efficacy of these vaccines in humans.

## 4. Reactivation of p53

The dysfunction of p53 is strongly implicated in the development of HPV-related cancers, with various mechanisms contributing to this impairment. These mechanisms include the following: p53 mutations, aberrant expression, single nucleotide polymorphisms (SNPs), post-translational modification, loss of heterozygosity, and sequestration by viral antigens [21]. Both in vitro and in vivo studies have shown that exogenous introduction of wild-type p53 into cervical cancers inhibits proliferation and triggers apoptosis [22]. As such, restoration of p53 function would appear to be a promising therapeutic strategy. Based on preclinical studies, several methods have been shown to enhance p53 function in cervical cancer. These include direct introduction of recombinant proteins [23], reactivation of p53 by intracellular factors [24], and using certain chemical compounds to raise p53 levels [25]. Interestingly, a clinical trial showed that, compared to chemotherapy alone, the addition of a recombinant human adenovirus-p53 (Ad-p53) to standard chemotherapy led to a significant reduction in tumor size in locally advanced cervical cancer [23]. Nevertheless, more clinical trials are needed to determine the utility of p53 reactivation in HPV-related cancers.

## 5. Oncolytic Viruses

Oncolytic viruses (OVs) are engineered viruses that can selectively induce immunogenic cell death in cancer cells while avoiding damaging normal cells [26]. Initially thought to kill cells through direct tumor lysis [27], the antitumor effects of OVs have been demonstrated to occur through a wide array of mechanisms, including enhancing innate and adaptive immune responses, altering the tumor microenvironment, and by transferring foreign genes to express pro-inflammatory cytokines [28]. Several preclinical studies have evaluated the therapeutic potential of OVs in HPV-related cancers which are briefly reviewed here. While the initial results are very promising, future clinical trials are needed to analyze the safety and effectiveness of these OVs in humans. OVs studied in HPV-related cancers include adenoviruses, herpes simplex viruses (HSV), parvoviruses, and the Newcastle disease virus.

### 5.1. Adenoviruses

Conditionally replicative adenoviruses (CRAd) are particularly appealing as therapeutic agents due to their ability to replicate selectively within specific target cells [29]. Notably, both adenoviruses and the HPV E6/E7 oncoproteins interact with similar regulatory proteins to influence the cell cycle. Specifically, the E1A protein of the adenovirus, which drives the adenoviral replication, has been shown to inhibit the p53 and the Rb tumor suppressor pathways [30,31]. As a result, CRAds with genomic deletions in the E1 region, which are designed to target HPV-positive tumors, have attracted clinical interest. Some studies have demonstrated significant oncolytic potential against both in vitro and in vivo models of HPV-positive HNSCC and cervical cancer [32,33].

### 5.2. Herpes Simplex Virus

HSVs possess various mechanisms to evade the immune system, making it an attractive candidate for genetic modification and use as a potent anti-tumor agent targeting cancer cells [34]. The therapeutic efficacy of a triple-mutated oncolytic HSV (T-01) in HPV-positive cervical cancer was studied by Kagabu et al. [35]. T-01 demonstrated significant cytotoxic activity against in vitro and in vivo models of HPV-positive cervical cancer. Interestingly, the study showed T-01 conferred increased major histocompatibility complex (MHC) class I expression in infected human cells. Additionally, there were increased numbers of CD8+ T cell precursor cells in tumor-bearing mice treated with T-01. This suggests the cytotoxic effects of T-01 against HPV-related cancer may have been driven by an immunomodulatory mechanism.

### 5.3. Parvoviruses

The parvovirus genome is composed of a single-stranded DNA, containing two promoters, P4 and P38. These promoters regulate the expression of the viral nonstructural proteins (NS1 and NS2) and the capsid proteins (VP1 and VP2), respectively [36]. The rat oncolytic H-1 parvovirus (H-1PV) demonstrated antineoplastic properties in various in vitro cell systems and animal models [37]. A study by Li et al. showed that co-treatment of cervical cancer cells with H-1PV and valproic acid, a histone deacetylase inhibitor, achieves synergy in killing cervical cancer cells [38]. At a molecular level, valproic acid causes the acetylation of parvovirus NS1 protein which modulates NS1-mediated cytotoxicity [38].

### 5.4. Newcastle Disease Virus

Oncolytic non-human viruses that are non-pathogenic in mammals have attracted interest in recent years. Newcastle disease virus (NDV) is a naturally occurring OV which causes severe illness in birds but not mammals [39]. In an in vitro study by Keshavars et al., NDV treatment suppressed the growth of HPV-associated cervical cancer cells by inducing apoptotic cell death. This effect was mediated by the production of reactive oxygen species [40]. Interestingly, localized oncolytic virotherapy with NDV has been shown to overcome systemic tumor resistance to immune checkpoint blockade immunotherapy. The immunostimulatory impact of NDV was especially significant when virotherapy was combined with immune checkpoint blockade using an anti-CTLA-4 antibody [41]. These findings have made NVD a possible therapeutic candidate as a selective antineoplastic agent for the treatment of HPV-associated cervical cancer.

## 6. Pattern Recognition Receptor Agonists

Pattern recognition receptors (PRR) are effectors of the innate immune system. By recognizing highly conserved pathogen-associated molecular patterns (PAMPs) such as lipopolysaccharides released by pathogens or danger-associated molecular patterns (DAMPs) released by damaged tissue, PRRs can induce the production of pro-inflammatory cytokines and activate the adaptive immune system [42]. PRRs include toll-like receptors (TLRs), nucleotide oligomerization domain-like receptors (NLRs), and retinoic acid-inducible-geneI (RIG-I)-like receptors, among others [43]. Tumor microenvironments, including in HPV-related cancers, exhibit dysregulated PRR expression, with activation of PRRs such as TLRs playing a role in inflammation, angiogenesis, and tissue invasion [43]. HPV is a potent inhibitor of PRRs, with HPV18 E7 having been shown to inhibit PRR STING-based NF-KB signaling and disrupt type I interferon secretion, inducing immune escape [44]. PRR agonists have been investigated in cancer therapy as vaccine adjuvants or for intra-tumoral delivery and are potential targets in HPV-related carcinomas as well. By increasing pro-inflammatory cytokine and chemokine synthesis, PRR agonists can activate tumor-infiltrating immune cells and induce tumor apoptosis [45].

In HPV-associated disease, the TLR7 agonist imiquimod is commonly prescribed for the treatment of genital warts. In two patients with refractory HPV-associated gynecological SCC, topical applications of imiquimod with intra-tumoral HPV-L1 vaccination (Gardasil) shortly after radiation therapy resulted in complete tumor responses [46]. A recent study by Cigno et al. [44] showed that activation of the anti-viral RNA-sensing RIG-I by the RIG-I agonist M8 promoted tumor growth arrest and apoptosis in CaSki and HeLa cells. PRR agonists such as STING agonists can potentially potentiate checkpoint inhibitors by creating an antitumor microenvironment [44]. Topical or intra-tumoral administration of PRR agonists have already shown efficacy in HPV-related disease, with continued investigation into their ability to alter the tumor microenvironment towards a more antitumor state.

## 7. Gene Silencing

HPV E6/E7 present distinct targets for gene silencing. Recent research investigating gene silencing with non-coding RNAs and CRISPR/Cas9 has shown promise in reversing E6/E7 oncogenic activity.

### 7.1. Non-Coding RNAs and RNA Interference

Non-coding RNAs such as small interfering RNAs (siRNAs) and microRNAs (miRNA) are double-stranded post-transcription regulators of gene expression, suppressing protein translation by degrading mRNA in a process known as RNA interference (RNAi). Synthetic non-coding RNAs first gained regulatory approval in 2018, when the FDA approved the siRNA agent patisiran in the treatment of hereditary transthyretin amyloidosis [47]. RNAi of E6/E7 has been investigated in HPV-related cancers as early as 1999. In 2003, inhibition of HPV18 E6 protein by Butz et al. [48] was shown to induce apoptosis in HPV-positive SiHa cells. siRNA designed to target the common promoter sequence for HPV16 E6/E7 decreased E6 and E7 mRNA by 45% and 50%, respectively, and E6/E7 protein expression by 30% [49]. In vitro siRNA-treated SiHa cells have shown decreased proliferation and increased rates of apoptosis. In vivo studies with non-coding RNAs have been conducted in mice to help determine optimal delivery platforms for non-coding RNAs. In a recent study, Deng et al. [50] showed that transdermal peptides could effectively deliver HPV16 L1, E6, and E7 siRNAs to SiHa xenograft tumors in mice, reducing the size of engrafted tumors and offering a potential pathway for application in humans.

There are no current clinical trials for non-coding RNAs in HPV-associated cancers. However, miRNA and recently characterized piwi-interacting RNAs (piRNAs), which suppress transposons and regulate genome stability, have been investigated as potential prognostic biomarkers in HPV-related HNSCC [51]. The expression of 305 piRNAs in Firmino et al. [51] revealed notable differences in expression between HPV-positive and HPV-negative HNSCC. piRNA expression signatures were also able to differentiate patients based on overall survival. Non-coding RNAs do not interfere with the host genome as their interactions occur post-transcription.

### 7.2. TALEN and CRISPR/Cas9

The development of the CRISPR/Cas9 (clustered regularly interspaced short palindrome repeats) system has allowed for highly specific gene editing and splicing. CRISPR gene therapies were recently approved in late 2023 by the EMA, FDA, and UK MHRA for treatment of sickle cell disease and transfusion-dependent beta-thalassemia [52]. CRISPR/Cas9 therapeutic potential in HPV-related disease was investigated as early as 2014 to induce selectively double-strand breaks in HPV E6 and E7 oncogenes [53]. These early in vivo studies showed that inactivating E6 and E7 oncogenes restored p53 and p21 function, inducing apoptosis in HPV-positive cervical cells [54]. Delivery vehicles for CRISPR/Cas9 have also been studied in vivo, with liposomes facilitating CD8+ T-cell infiltration in cervical cancer cells and upregulating pro-inflammatory cytokines [55]. Jubair et al. [56] used the same liposomes developed for siRNAs in CRISPR/Cas9 delivery, protecting CRISPR guide RNA against serum nucleases for intravenous injection in mice with cervical cancer xenografts. Mice treated with CRISPR/Cas9 subsequently showed higher levels of cleaved caspase-3 protein, a marker of apoptosis.

Other gene-editing modalities include transcription-activator-like effector nucleases (TALEN), engineered restriction enzymes that also selectively induce double-strand breaks for gene editing. TALEN proteins [57] are composed of a customizable DNA-binding domain attached to a Fok1 nuclease [58]. The central DNA-binding domain consists of a variable number of 33–35 amino acid units, with variable amino acids at positions 12 and 13 conferring specificity in nucleotide binding [59]. TALENs have been designed to target HPV16 E7, triggering necrosis in SiHa cervical cancer cells [60]. HPV16 E7 TALENs have been shown to reduce tumorigenesis when injected subcutaneously in HPV16 positive transgenic mice, and topical cervical application of TALENs reduced HPV16 DNA load in the same mice [61].

Clinical trials for TALEN and CRISPR/Cas9 in the treatment of HPV include a study of TALEN and CRISPR/Cas9 in Sun Yat-Sen University in China, although no other clinical trials were found per review. TALENs are generally more specific than CRISPR/Cas9 platforms because they tolerate less mismatches compared to CRISPR/Cas9 platforms, whereas CRISPR/Cas9 is more efficient [62]. CRISPR/Cas9 platforms are easier to synthesize with guide RNA compared to the protein constructs of TALENs. Compared to RNA interference, which only temporarily blocks E6/E7 translation, TALENs and CRISPR potentially offer more durable antitumor effects by disrupting integrated HPV DNA in cell nuclei [61].

Practical challenges to deploying gene editing technologies include costs of development, off-target editing, and immunogenicity of gene editing systems [62]. Because E6/E7 are relatively small oncogenes of 500 and 300 base pairs, respectively, the risk of off-target editing is higher [56]. CRISPR/Cas9 has been shown to tolerate a number of base pair mismatches that can result in off-target editing [56]. Determining the acceptable risk of off-target mutations, which can in turn potentially cause cancer, will be crucial, although in vivo clinical trials for the application of CRISPR/Cas9 and TALENs in patients with cancer remain limited. CRISPR/Cas9 trials have primarily focused on gene editing of ex vivo T-lymphocytes [63].

Gene editing raises multiple ethical issues. Although most ethical considerations of gene editing are related to the long-term effects of germline editing, concerns surrounding somatic gene editing include healthcare equity and justice. Due to large inter and intranational disparities in healthcare access, there are concerns that only the wealthiest individuals in the wealthiest nations will benefit from gene editing given the immense cost of development [64]. For example, the cost of the recently FDA-approved sickle cell treatment Lyfgenia exceeds 3.1 million USD [65].

## 8. Adoptive Cell Transfer

Cellular therapies such as chimeric antigen receptor T-cell (CAR-T) therapy have improved the outcomes of refractory B-cell lymphomas. Lifileucel, a tumor-infiltrating lymphocyte (TIL) immunotherapy, has been approved by the FDA in the treatment of refractory unresectable or metastatic melanoma [66]. It is the first cellular therapy approved in solid tumors. Besides melanoma, adoptive cell transfer (ACT) therapies including CAR-T, TIL, and engineered T-cell receptors are being developed in other solid malignancies, including recurrent/refractory or metastatic HPV-positive cancers [67]. E6 and E7 are antigen targets in HPV-positive cancers that can be utilized to develop engineered T-cell receptors (TCRs) and CARs.

### 8.1. Tumor-Infiltrating Lymphocytes

Highly immunogenic tumors such as melanoma often have significant tumor lymphocyte infiltration [68]. These tumor infiltrating lymphocytes (TILs) include CD4 and CD8 T-cells and can be isolated from resected tumors, cultured and expanded in the presence of high-dose IL-2, and re-infused into patients after lymphodepleting chemotherapy. TILs can be expanded in bulk or selected for tumor-reactive lymphocytes [69]. Bulk expansion of TILs may be beneficial in recognizing multiple tumor neoantigens but potentially include non-reactive T-cells and Tregs.

In a study of 274 patients with oropharyngeal cancers, high levels of TILs were associated with improved survival [70]. TIL levels were correlated with HPV-positive disease, which generally has better prognosis than HPV-negative oropharyngeal cancer. In HPV-positive tumors with low TILs, survival was comparable to HPV-negative tumors, suggesting that increased immunogenicity of HPV-positive drives increased survival compared to HPV-negative oropharyngeal cancer. Clinical trials for TILs in HPV-related cancers have shown modest efficacy. A Phase II trial of TILs selected for HPV16/18 E6 and E7 reactivity in 29 patients with metastatic HPV-associated epithelial cancers showed objective responses in 5/18 patients with cervical cancer and 2/11 patients with other HPV-associated cancers [71]. Two patients in the cervical cancer cohort achieved complete and durable responses. No toxicities were related to infusions or to autoimmune adverse events, with most toxicities attributed to lymphocyte conditioning and the IL2 analog aldesleukin. Another Phase I study investigated the safety of TILs for treatment of cervical cancer in patients after standard chemoradiation therapy and found tolerable AEs, although infusions were disrupted by the COVID-19 pandemic and insufficient T-cell isolation and expansion [72]. TILs can be more easily procured compared to engineered T-cell therapies, although their efficacy remains modest in HPV-related cancers.

### 8.2. Engineered T-Cell Therapy

Engineered T-cell therapies include engineered T-cell receptor therapies and chimeric antigen receptor T-cell (CAR-T) therapy. T-cell receptor therapies are gaining traction in the treatment of solid tumors due to their larger range of antigenic targets, as they can target antigens presented by major histocompatibility complexes (MHCs), and due to their lower thresholds of activation compared to CAR-T cells [73]. As with other ACT therapies, T-cells are first isolated from patients’ tumors or health donors’ blood. They are subsequently stimulated with target antigens and clonally expanded before being reintroduced by infusion into patients [73]. TCRs can be genetically edited to select for specific antigens such as NY-ESO-1 (New York esophageal SCC 1) after isolating reactive T cells and cloning TCR genes into a retroviral vector backbone, with such therapies showing efficacy in melanoma [74].

Engineered TCRs against E6 have shown promise in a Phase I/II clinical trial of 12 patients with metastatic HPV-positive cervical, anal, oropharyngeal, and vaginal cancer treated with TCR T-cells and the IL-2 analog aldesleukin [67]. Patients numbering 2/12 achieved objective tumor responses, with one patient showing durable response to therapy with no evidence of disease after 3 years of treatment. No autoimmune adverse events or off-target toxicities were noted, with cytopenias due to lymphocyte-depleting conditioning being the most common toxicity. The modest overall response to treatment was attributed to tumor escape mechanisms, including HLA loss and expression of PD-1/PD-L1. Similarly, genetically engineered TCRs targeting HPV16 E7 showed efficacy in the treatment of HPV-positive uterine, cervical, anal, and head and neck cancer, with 6/12 patients showing objective tumor regression and 3/12 patients showing continued durable tumor regression [75]. Infused engineered T-cells persisted in peripheral blood and continued to have reactivity against E7. Biopsy of relapsed tumors showed that HLA loss may have contributed to lack of efficacy or relapse. Similar to the prior study, most toxicities were cytopenias related to conditioning preceding lymphocyte depletion. TCR clinical trials are ongoing in HPV-related disease, including a Phase II trial investigating HPV E7 TCRs with aldesleukin [76]. Trials are also investigating TCR therapy with concurrent immune checkpoint inhibitors (Table 1).

CAR-T therapy has also been studied in HPV, with selective CARs developed against E6 [77]. No clinical trials have started for CAR-T therapy in HPV-related cancers, although Phase I/II trials have investigated its safety and efficacy in solid tumors, with limited responses [78,79]. Challenges to TCR and CAR-T therapy in general include finding antigen targets, tumor immune escape, significant off-target toxicities, and costs of therapy [73]. Compared to CAR-T therapy, TCRs are restricted to patients with specific HLA alleles, as TCRs must be manufactured to recognize antigens presented on corresponding MHCs. While CAR-T cells utilize antibody-based targeting, TCRs recognize antigens expressed by host MHC complexes. Cell models in hematological malignancies have suggested that CAR-T cells have greater initial effector potency but may be unable to sustain differentiation and efficacy compared to TCR T-cells, although functional differences remain poorly defined [80].

## 9. HPV-Related Cancers and Circulating Tumor DNA

Current surveillance methods for the detection of recurrence and response to treatment in HPV-related cancers consist of history-taking, physical examination, and surveillance imaging with decreasing frequency. However, recurrences are often clinically silent [81] and detected via imaging or diagnosed when patients report symptoms, with only a minority identified during routine surveillance physical examinations. Biologic markers have the potential to assess treatment response and predict recurrence in HPV-related cancers [82].

HPV ctDNA comprises fragments of the double-stranded HPV genome released into the bloodstream or other bodily fluids following tumor cell death [83]. When amplified using techniques like polymerase chain reaction (PCR), their detection can predict early recurrence, aid in the postoperative treatment plan, and as a marker of response to treatment [84]. ctHPV DNA has already been validated as a minimally invasive biomarker for HPV-related anal [85], cervical cancers [86], and oropharyngeal cancers [87]. A significant challenge in using ct-DNA is distinguishing between tumor DNA and host DNA. There are limitations in using ctDNA. For example, conventional PCR methods show variable detection rates for serum HPV DNA: 12–45% in cervical cancer patients and 19–79% in oropharyngeal cancer patients [83]. A recent trial showed that while there is a correlation with therapy response, approximately 10% of patients with HPV-related oropharyngeal cancer did not have detectable levels of ctDNA at diagnosis [88]. Overall, ctDNA remains an evolving field. More research and clinical trials are needed before these biomarkers can be reliably incorporated into cancer diagnosis and surveillance schedule.

## 10. Current Immunotherapy in HPV-Positive Disease

Immune checkpoint inhibitors (ICIs), including anti-PD-1/PD-L1, anti-CTLA4, and anti-LAG3 antibodies, have become a mainstay of cancer therapy in the last decade, including in HPV-positive disease [89]. ICIs remove immune checkpoint brakes on T-lymphocytes from transmembrane protein signaling, which cancer cells utilize to induce T-cell exhaustion [90]. ICIs can thus restore immune antitumor responses.

In head and neck SCC (HNSCC), the anti-PD1 antibody pembrolizumab has been approved by the FDA as monotherapy or in combination with chemotherapy for the treatment of metastatic or unresectable PD-L1 positive disease [91]. Approval was based on the KEYNOTE-048 trial, which showed improved median OS for patients who received pembrolizumab + chemotherapy compared to cetuximab (an EGFR inhibitor) + chemotherapy [91,92]. HPV-positive HNSCC is associated with increased immunogenicity and response to checkpoint inhibitor therapy [93]. Neoadjuvant immunotherapy is also being studied in HNSCC [91].

In cervical cancers, HPV16 E7 protein has been shown to induce greater PD-L1 expression in infected cells [94]. The FDA approved single-agent treatment with pembrolizumab in patients with recurrent or metastatic cervical cancer with disease progression on or after chemotherapy and positive tumor PD-L1 expression (CPS score > 1%) in 2018 [95]. In 2021, the FDA further approved pembrolizumab in combination with chemotherapy, with or without bevacizumab, for patients with persistent, recurrent, or metastatic cervical cancer (with CPS ≥ 1) [96]. Most recently, in January 2024, pembrolizumab with chemoradiation was approved for patients with FIGO 2014 Stage III–IVa cervical cancer based on the Phase III KEYNOTE-A18 trial [97]. In Canada and Europe, the anti-PD1 inhibitor cemiplimab has been approved for advanced cervical cancer patients with progression after first-line platinum-containing therapy in March and November 2022, respectively [98].

Pembrolizumab is also being investigated in the treatment of anal SCC, with promising results [99], as well as for rarer HPV-positive diseases such as vaginal and vulvar SCC [100].

## 11. Conclusions and Future Directions

HPV-positive cancers present unique targets for therapy due to the key roles of E6 and E7 in driving oncogenesis, as well as the high immunogenicity of HPV-positive cancers. Inhibition of E6 and E7 has been shown to reverse HPV-related oncogenesis. Currently, immunotherapy with immune checkpoint inhibitors has already changed the treatment landscape in HNSCC and cervical cancer, with further investigation into its efficacy in anal SCC. Future directions beyond immune checkpoint inhibitor therapy include other ways of generating antitumor immune responses, including cell therapies, PRR agonists, oncolytic adenoviruses, and therapeutic cancer vaccination. Clinical trials for cellular therapies are underway in HPV-positive local or metastatic tumors, including T-cell receptors targeting E6/E7 and tumor-infiltrating lymphocytes. For PRR agonists, imiquimod has already been used to treat HPV-related anogenital warts and has been investigated in the treatment of HPV-related gynecological SCC. Other PRR-agonists are also being studied in cancer therapy, especially in their role as potentiators of immune checkpoint inhibitor therapy through their mediation of the tumor microenvironment. Similarly, oncolytic adenoviruses were previously thought to exert direct antitumor effects, although new studies have shown that they have complex interactions with the immune system and the tumor microenvironment. Lastly, therapeutic cancer vaccines have been studied in HPV-positive disease to induce immunogenicity against tumors. Although efficacy remains limited, the development of more effective antigen delivery systems for cancer vaccines has shown promise in animal models.

Outside of immunotherapy, other potential treatment modalities include gene silencing (such as with RNAi, TALENs, and CRISPR), with the potential for local topical applications. Clinical trials are needed to determine the efficacy and feasibility of these new technologies in the treatment of HPV-related cancers.

## Figures and Tables

**Table 1:** Representative adoptive cell transfer clinical trials.

Identifier	Phase	Enrollment	Eligibility	Target (If Applicable)	Concurrent Therapies	Country
Tumor-infiltrating lymphocyte therapy						
NCT01585428	II	29	HPV 16/18 + ve metastatic or locally advanced tumors	-	IL-2 analog	USA
NCT02421640	II	116 (estimated)	Nasopharyngeal carcinoma	-	Cisplatin	China
NCT05475847	I	20 (estimated)	Persistent, recurrent, or metastatic cervical cancer	-	-	China
NCT03108495	II	189 (estimated)	Persistent, recurrent, or metastatic cervical cancer	-	Pembrolizumab (in one cohort)	
T-cell receptor therapy						
NCT02858310	I/II	180 (estimated)	HPV-16 +v e metastatic or refractory cancer, HLA-A * 02:01 allele	E6	IL-2 analog	USA
NCT05639972	I/II	15 (estimated)	HPV-16 + ve locally advanced tumor, HLA-A * 02:01 allele	E7	IL-2 analog	USA
NCT05686226	II	20 (estimated)	HPV-16 + ve metastatic or recurrent cancer, HLA-A * 02:01 allele	E7	IL-2 analog	USA
NCT06358053	I	24 (estimated)	HPV + ve metastatic or recurrent cancer	E6	IL-2 analog	China
NCT05122221	I	12 (estimated)	HPV 16 + cancer, HLA-A * 02:01 allele	E7	*-*	China
NCT05357027	I/II	18 (estimated)	Persistent, recurrent or metastatic cervical cancer	E6	IL-2 analog	China
